# Association between triglyceride glucose index and worsening heart failure in significant secondary mitral regurgitation following percutaneous coronary intervention

**DOI:** 10.1186/s12933-022-01680-9

**Published:** 2022-11-28

**Authors:** Haozhang Huang, Qiang Li, Jiulin Liu, Linfang Qiao, Shiqun Chen, Wenguang Lai, Yu Kang, Xiaozhao Lu, Yang Zhou, Yibo He, Jiyan Chen, Ning Tan, Jin Liu, Yong Liu

**Affiliations:** 1 Department of Cardiology, Guangdong Cardiovascular Institute, Guangdong Provincial People’s Hospital, Guangdong Academy of Medical Sciences, Guangzhou, 510080 China; 2 Department of Guangdong Provincial Key Laboratory of Coronary Heart Disease Prevention, Guangdong Cardiovascular Institute, Guangdong Provincial People’s Hospital, Guangdong Academy of Medical Sciences, Guangzhou, 510080 China; 3 The Second School of Clinical Medicine, Southern Medical University, Guangzhou, 510515 China

**Keywords:** Triglyceride glucose index, Heart failure, Secondary mitral regurgitation, Percutaneous coronary intervention

## Abstract

**Background:**

The triglyceride glucose (TyG) index is an alternative to insulin resistance (IR) as an early indicator of worsening heart failure (HF). Patients with secondary mitral regurgitation (sMR) often experience progressive deterioration of cardiac function. This study aimed to investigate the relationship between the TyG index and worsening of HF in significant sMR (grade  ≥ 2) following percutaneous coronary intervention (PCI).

**Methods:**

This study enrolled participants with significant sMR following PCI from a multicenter cohort study. The patients were divided into the following 3 groups according to tertiles of TyG index: T1, TyG ≤ 8.51; T2, TyG > 8.51 to ≤ 8.98; and T3, TyG > 8.98. The main clinical outcome was worsening HF including unplanned rehospitalization or unscheduled physician office/emergency department visit due to HF and unplanned mitral valve surgery.

**Results:**

A total of 922 patients (mean ± SD age, 64.1 ± 11.0 years; 79.6% male) were enrolled. The incidence of worsening HF was 15.5% in T1, 15.7% in T2, and 26.4% in T3. In the multivariable model, the highest TyG tertile (T3 group) was more strongly correlated with worsening HF than the lowest tertile (T1 group) after adjusting for confounders (adjusted hazard ratio, 2.44; 95% confidence interval, 1.59–3.72; P < 0.001). The addition of TyG to risk factors such as N-terminal pro brain natriuretic peptide and clinical models improved the predictive ability of TyG for worsening HF.

**Conclusions:**

Elevated preprocedural TyG index is a significant and independent risk factor for worsening HF in sMR following PCI that can be used for risk stratification.

**Supplementary Information:**

The online version contains supplementary material available at 10.1186/s12933-022-01680-9.

## Introduction

Secondary mitral regurgitation (sMR), a frequent complication of coronary artery disease (CAD) following percutaneous coronary intervention (PCI), can cause progressive deterioration of cardiac function, ultimately resulting in worsening heart failure (HF). Around 10% of patients with CAD have moderate or severe sMR due to secondary ischemia [1–5].

Insulin resistance (IR), a marker of metabolic disorder and inflammation, is a well-established independent risk factor for worsening HF [6, 7]. The triglyceride glucose (TyG) index is a simple, cost-effective, and reliable surrogate marker for IR, and numerous clinical studies have shown that it is associated with cardiovascular disease (CVD) and the incidence of worsening HF or major adverse cardiovascular events (MACE) in the general population [8, 9] as well as in patients with stable CAD [10, 11], acute coronary syndrome [12, 13], and HF [14, 15]. However, the clinical significance of the TyG index for worsening HF in significant sMR is not known.

The present study investigated the clinical utility of TyG for predicting worsening HF in patients with significant sMR following PCI by analyzing the relationship between these 2 variables in a patient cohort from 5 different medical centers.

## Methods

### Study population

In this multicenter cohort study, we analyzed data from Cardiorenal Improvement II (CIN-II), an observational, multicenter trial of patients treated at 5 large tertiary hospitals (ClinicalTrials.gov NCT05050877) in Southern China between January 2007 and December 2020. The indication for PCI or coronary angiography was based on signs or symptoms of ischemia, elevated diagnostic enzymes, or abnormal electrocardiogram findings, and the procedures were performed in accordance with standard clinical practice guidelines [16, 17].

**Fig. 1:**
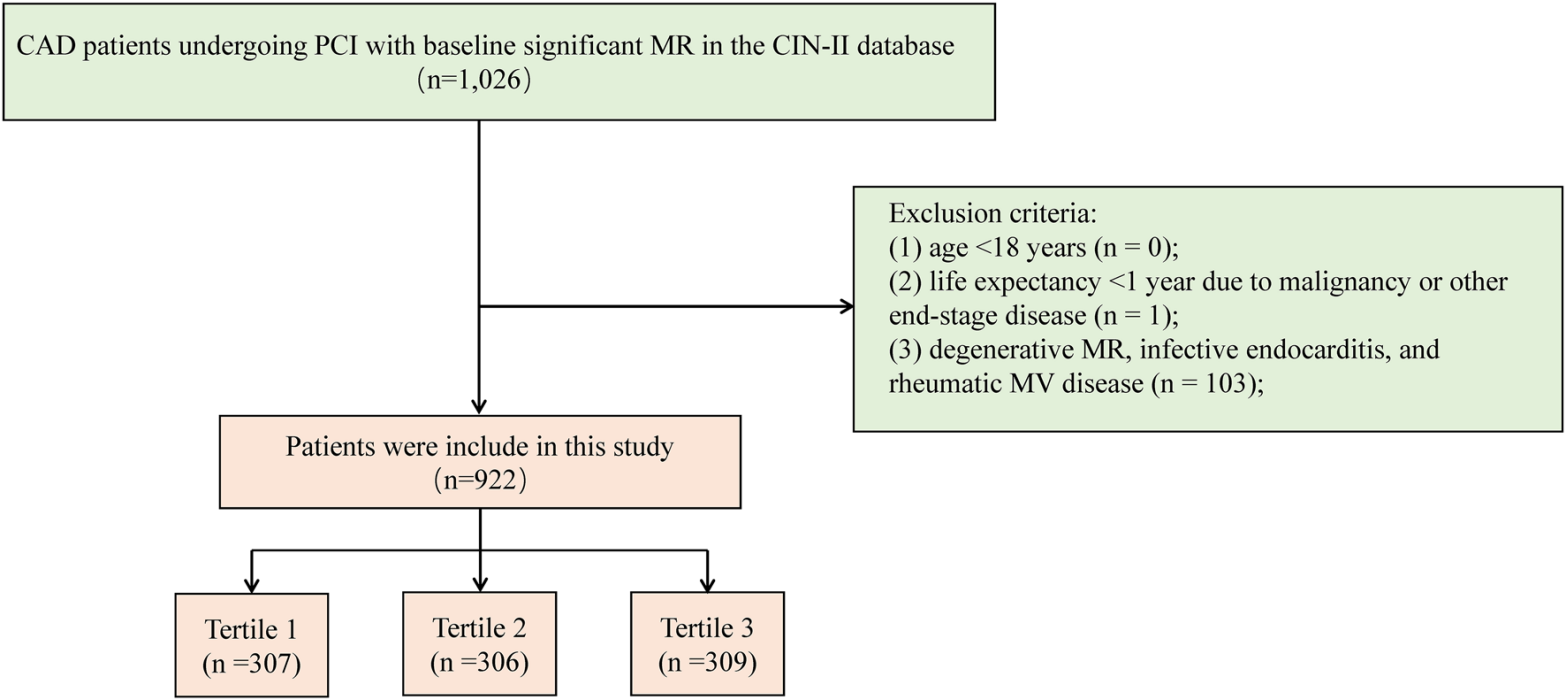
Flow diagram of patient selection

A total of 1026 CAD patients with moderate or severe MR who underwent PCI upon admission were identified in the CIN-II database. The exclusion criteria were as follows: (1) age < 18 years (n = 0); (2) life expectancy  < 1 year due to malignancy or other end-stage disease (n = 1); and (3) degenerative MR, infective endocarditis, or rheumatic mitral valve disease (n = 103). Ultimately, 922 patients with moderate or severe sMR following PCI were included in the study (Fig. 1).

### Definitions

Echocardiography data were extracted from the electronic medical record system, recorded by trained sonographers following the same standard, and interpreted by experienced cardiologists at the Echocardiography Reading Center of Guangdong Provincial People’s Hospital. The presence of MR was determined on the first echocardiography examination, which was generally performed within 48 h of admission. The severity of MR, which was evaluated by visual assessment integrating Doppler echocardiography data from multiple acoustic windows and incorporating qualitative and semiquantitative methods, was divided into the following 5 levels: none = 0, mild = 1, moderate = 2, moderately severe = 3, or severe = 4. Significant MR was defined as grade  ≥ 2. Degenerative MR was defined by the mitral valve morphologic descriptors of abnormal, myxomatous, flail, prolapsed, or thickened valves. In the absence of degenerative MR or rheumatic mitral valve disease, MR was classified as secondary.

TyG was calculated using the following equation: TyG = ln (fasting triglyceride [TG, mg/dl] × fasting plasma glucose [FPG, mg/dl]/2). After an overnight fast, venous samples were typically obtained before PCI via aseptic technique for plasma glucose and lipid measurements. FPG was determined with the glucose oxidase method. TG was determined by an enzymatic method. If the patient had undergone emergency PCI, the first available FPG measurement after the procedure was used.

Chronic kidney disease (CKD) was defined as an estimated glomerular filtration rate (eGFR) calculated using the Chronic Kidney Disease Epidemiology Collaboration value of  < 60 ml/min/1.73m^2^ [18]. Congestive heart failure (CHF) was defined as New York Heart Association class  > 2 or Killip class  > 1. Anemia was defined as a hematocrit  ≤ 39% for males or  ≤ 36% for females. Hyperlipidemia was defined as fasting total cholesterol ≥ 240 mg/dl, low-density lipoprotein cholesterol  > 160 mg/dl, TG ≥ 150 mg/dl, or high-density lipoprotein cholesterol  < 40 mg/dl. Diabetes was categorized as either known diabetes (defined as ongoing medical treatment for diabetes [insulin or antidiabetics]) or newly diagnosed diabetes (defined as hemoglobin A1c level  ≥ 6.5%).

### Study endpoints

The primary endpoint of the study was worsening HF, which was defined as unplanned rehospitalization or unscheduled physician office/emergency department visits due to HF and unplanned mitral valve surgery. The secondary endpoints were long-term all-cause mortality, long-term cardiovascular-specific mortality, and major adverse cardiovascular events (MACE; i.e., cardiovascular-specific mortality, nonfatal acute myocardial infarction [MI], or nonfatal stroke). During follow-up, nonfatal MI was determined based on electrocardiography, cardiac enzyme values, and typical symptoms. Nonfatal stroke was defined as nonfatal cerebral infarction, intracerebral hemorrhage, and unspecified stroke. Cardiovascular-specific death was defined as death due to MI, sudden cardiac death, HF, stroke, cardiovascular procedures, and other cardiovascular causes. Follow-up was conducted either face-to-face or by telephone. All-cause mortality, cardiovascular mortality (International Classification of Diseases, 10th Revision [ICD-10] codes I00–I99 for underlying cause of death), and noncardiovascular mortality (all other ICD-10 codes for underlying cause of death) were also evaluated.

### Statistical analysis

Patients were divided into tertiles according to TyG index—i.e., tertile 1 (T1), TyG ≤ 8.51; T2, TyG > 8.51 to  ≤ 8.98; and T3, TyG > 8.98. Descriptive statistics are reported as the mean with standard deviation (SD), median with interquartile range (IQR), or number and percentage when appropriate. The chi-squared test was used to evaluate differences between categorical variables. The independent samples Student’s t test was used to compare continuous variables with normal distribution, and the Mann–Whitney U test was used to compare non-normally distributed continuous variables. A Bonferroni-corrected P value was calculated as the P value multiplied by the number of tests (n = 3).

A restricted cubic spline analysis was performed to determine the dose–response relationship between TyG index and risk of the primary endpoint. Endpoints were assessed with the Kaplan–Meier method and compared with the log-rank test. Independent associations between TG, FPG, TG/FPG, and TyG index and endpoints of interest were assessed with a Cox regression model and are expressed as the adjusted hazard ratio (HR) with a 95% confidence interval (CI). Covariates were selected based on prior literature and clinical experience [5, 14, 19]. Model 1 was unadjusted. Model 2 was adjusted for age (as a continuous variable), gender, and left ventricular ejection fraction (LVEF). Model 3 included age; gender; smoking history; body mass index (BMI); LVEF; hyperlipidemia; hypertension; diabetes mellitus (DM); anemia; CKD; acute MI and atrial fibrillation (AF); and renin–angiotensin–aldosterone system inhibitor, beta-blockers, loop diuretics, and mineralocorticoid receptor antagonist. We also conducted subgroup analyses of patients stratified by age (≤ 65 vs > 65 years), gender (male vs female), BMI (< 25 vs ≥ 25 kg/m^2^), CHF (no vs yes), and acute coronary syndrome (ACS) (no vs yes). Interactions between subgroups were assessed with the likelihood ratio test.

Net reclassification improvement (NRI; with risk categories set at  < 10%, 10–30%, and  > 30%), integrated discrimination improvement (IDI), and the difference in Harrell’s C statistic were calculated to assess reclassification and discrimination improvements achieved by adding TG, FPG, TG/FPG, and the TyG index to commonly used clinical indices (N-terminal pro brain natriuretic peptide [NT-proBNP], LVEF, and high-sensitivity troponin T) and clinical models (Global Registry of Acute Coronary Events [GRACE] score and Muerte Subita en Insuficiencia Cardiaca Study [MUSIC] Risk score) [20, 21]. The GRACE score was calculated from 8 variables that are readily obtained at hospital admission (age, heart rate, systolic blood pressure, serum creatinine concentration, Killip class, cardiac arrest, presence of ST segment deviation, and elevated cardiac enzymes/markers). The MUSIC Risk score was calculated based on the following parameters: prior atherosclerotic vascular event, left atrial size  > 26 mm/m^2^, LVEF  ≤ 35%, AF, left bundle branch block or intraventricular conduction delay, nonsustained ventricular tachycardia and frequent ventricular premature beats, eGFR  < 60 ml/min/1.73 m^2^, hyponatremia  ≤ 138 mmol/L, NT-proBNP  > 1000 ng/L, and troponin-positivity.

All P values were 2-sided, and P < 0.05 was considered to indicate statistically significant differences. Statistical analyses were performed using R v4.1.3 (R Institute for Statistical Computing, Vienna, Austria).

## Results

### Baseline characteristics of the study population

A total of 922 patients who underwent PCI with significant sMR were included in the analysis; their clinical characteristics at baseline are shown in Table 1 and Additional file 1: Table S1. The mean age of the patients was 64.1 ± 11.0 years, and 20.4% were female (n = 188). Patients with higher TyG index were generally younger and more frequently female. Some high-risk complications were more common in the T3 group than in the T1 group such as DM (55.7 vs 23.3%, P < 0.001, Bonferroni-corrected P < 0.001) and CKD (42.0 vs 32.4%, P = 0.002; Bonferroni-corrected P = 0.006). Moreover, the T3 group had a lower LVEF and eGFR and were more likely to have higher ProBNP than the T1 group. The 3 groups did not differ in terms of the PCI procedure and were similar in most other baseline characteristics.

**Table 1:** Baseline Characteristics According to TyG index level

Characteristic	TyG index level				P-value
	Overall	T1 (≤ 8.51)	T2 (> 8.51, ≤ 8.98)	T3 (> 8.98)	
	N = 922	N = 307	N = 306	N = 309	
Demographic characteristics					
Male	734 (79.6)	261 (84.5)	240 (78.4)	233 (75.9)	0.025
Age, years	64.1 (11.0)	64.3 (11.4)	65.4 (11.0)	62.6 (10.5)	0.006
BMI, kg/m^2^	23.8 (3.4)	23.5 (3.4)	23.5 (3.3)	24.2 (3.6)	0.021
Heart rate, bmp	82.1 (17.4)	81.0 (16.6)	81.3 (18.6)	84.0 (16.7)	0.059
SBP, mmHg	128.1 (22.8)	126.9 (22.6)	128.7 (22.1)	128.6 (23.7)	0.566
DBP, mmHg	75.6 (12.9)	75.5 (13.4)	75.5 (12.2)	75.7 (13.2)	0.96
History of smoke					0.025
Never	567 (61.5)	185 (59.9)	180 (58.8)	202 (65.8)	
Cessation	139 (15.1)	59 (19.1)	49 (16.0)	31 (10.1)	
Current	216 (23.4)	65 (21.0)	77 (25.2)	74 (24.1)	
Medical history					
Anemia	404 (43.8)	145 (46.9)	134 (43.8)	125 (40.7)	0.299
CHF	416 (45.1)	131 (42.4)	139 (45.4)	146 (47.6)	0.433
DM	404 (43.8)	145 (46.9)	134 (43.8)	125 (40.7)	< 0.001
CKD	341 (37.0)	100 (32.4)	112 (36.6)	129 (42.0)	0.045
Hypertension	552 (59.9)	183 (59.2)	183 (59.8)	186 (60.6)	0.942
Hyperlipidemia	690 (74.8)	226 (73.1)	233 (76.1)	231 (75.2)	0.678
AF	105 (11.4)	52 (16.8)	26 (8.5)	27 (8.8)	0.001
COPD	29 (3.1)	10 (3.2)	11 (3.6)	8 (2.6)	0.777
Stroke	39 (4.2)	15 (4.9)	12 (3.9)	12 (3.9)	0.8
History of PCI	119 (12.9)	40 (12.9)	38 (12.4)	41 (13.4)	0.942
History of AMI	99 (10.7)	29 (9.4)	31 (10.1)	39 (12.7)	0.378
Clinical presentation					
AMI	343 (37.2)	101 (32.7)	116 (37.9)	126 (41.0)	0.095
STEMI	227 (24.6)	72 (23.3)	75 (24.5)	80 (26.1)	0.728
NSTEMI	116 (12.6)	29 (9.4)	41 (13.4)	46 (15.0)	0.097
Laboratory test					
LDL-C, mmol/L	2.88 (1.10)	2.77 (1.05)	2.95 (1.19)	2.91 (1.06)	0.117
HDL-C, mmol/L	0.97 (0.27)	0.98 (0.28)	0.97 (0.27)	0.96 (0.28)	0.75
eGFR, mL/min/1.73m^2^	69.6 (26.0)	73.5 (27.4)	68.6 (23.9)	66.8 (26.1)	0.004
ALB, g/L	35.0 (4.6)	34.5 (4.1)	35.3 (5.1)	35.1 (4.5)	0.088
NT-proBNP, ng/L	2092 [903, 4826]	2414 [1169, 5311]	1864 [860, 4606]	1739 [749, 4816]	0.04
hs-cTnT, ng/L	0.71 [0.22, 7.33]	0.64 [0.22, 4.20]	0.62 [0.22, 6.33]	1.13 [0.23, 9.89]	0.169
LVEF, %	43.8 (13.7)	42.8 (13.6)	45.5 (13.9)	43.2 (13.4)	0.029
LVEDD, mm	43.3 (10.7)	44.1 (10.8)	42.5 (10.6)	43.3 (10.5)	0.165
LVESD, mm	56.6 (8.5)	57.3 (8.7)	56.1 (8.5)	56.5 (8.4)	0.22
Left atrial, mm	41.2 (6.3)	41.7 (6.3)	41.0 (6.7)	41.1 (6.0)	0.309
Procedural characteristics					
Radial artery access	754 (81.8)	256 (82.8)	247 (80.7)	251 (81.8)	0.791
Multivessel disease	789 (85.6)	263 (85.1)	261 (85.3)	265 (86.3)	0.9
Culprit vessel in STEMIs					0.945
Left main coronary artery	7 (2.8)	1 (1.4)	3 (3.6)	3 (3.4)	
LAD	115 (46.6)	37 (50.0)	39 (46.4)	39 (43.8)	
LCX	41 (16.6)	11 (14.9)	13 (15.5)	17 (19.1)	
RCA	84 (34.0)	25 (33.8)	29 (34.5)	30 (33.7)	
Number of vessels treated	1.45 (0.68)	1.41 (0.67)	1.49 (0.68)	1.44 (0.69)	0.346
Number of lesions treated	1.67 (0.89)	1.63 (0.89)	1.70 (0.86)	1.68 (0.94)	0.62
Number of stents	1.91 (1.18)	1.92 (1.16)	1.92 (1.24)	1.90 (1.15)	0.96
Minimum stent diameter, mm	2.69 (0.75)	2.70 (0.77)	2.64 (0.80)	2.72 (0.69)	0.413
Lesion length, mm	52.3 (36.3)	52.5 (36.0)	52.5 (38.4)	52.0 (34.7)	0.977
Discharge prescription					
RAAS inhibitor	618 (67.0)	220 (71.2)	191 (62.4)	207 (67.4)	0.067
Beta-blockers	781 (84.7)	265 (85.8)	254 (83.0)	262 (85.3)	0.594
CCB	144 (15.6)	40 (12.9)	47 (15.4)	57 (18.6)	0.156
Statin	856 (92.8)	287 (92.9)	286 (93.5)	283 (92.2)	0.827
Aspirin	870 (94.4)	293 (94.8)	285 (93.1)	292 (95.1)	0.519
Clopidogrel	807 (87.5)	276 (89.3)	267 (87.3)	264 (86.0)	0.451
Loop diuretic	461 (50.0)	154 (49.8)	149 (48.7)	158 (51.5)	0.788
MRA	467 (50.7)	160 (51.8)	149 (48.7)	158 (51.5)	0.702
Hypoglycemic drugs	199 (21.9)	40 (13.0)	67 (22.3)	92 (30.7)	< 0.001
Insulin	40 (4.3)	10 (3.2)	10 (3.3)	20 (6.5)	0.072

Median (interquartile range). Lesion morphology assessed by operators

*AF* atrial fibrillation, *AMI* acute myocardial infarction, *ALB* albumin, *CHF* congestive heart failure, *CKD* chronic kidney disease, *COPD* chronic obstructive pulmonary disease, *CCB* calcium channel blockers, *DBP* diastolic blood pressure, *DM* diabetes mellitus, *eGFR* estimated glomerular filtration rate, *HDL-C* high-density lipoprotein cholesterol, *hs-cTnT* Hypersensitive troponin T, *LDL-C* low-density lipoprotein cholesterol, *LAD* left anterior descending coronary artery, *LCX* left circumflex coronary artery, *LVEF* left ventricular ejection fraction, *LVEDD* left ventricular end-diastolic dimension, *LVESD* left ventricular end-systolic dimension, *MRA* mineralcorticoid recept antagonist, *NSTEMI* non-ST-segment elevation myocardial infarction, *NT-proBNP* N-terminal pro brain natriuretic peptide, *TyG index* Triglyceride–glucose index, *PCI* percutaneous coronary intervention, *RAAS inhibitor* renin–angiotensin–aldosterone system inhibitor, *RCA* right coronary artery, *SBP* systolic blood pressure, *STEMI* ST-segment elevation myocardial infarction

### Predictive ability of TyG index for clinical outcomes

**Fig. 2:**
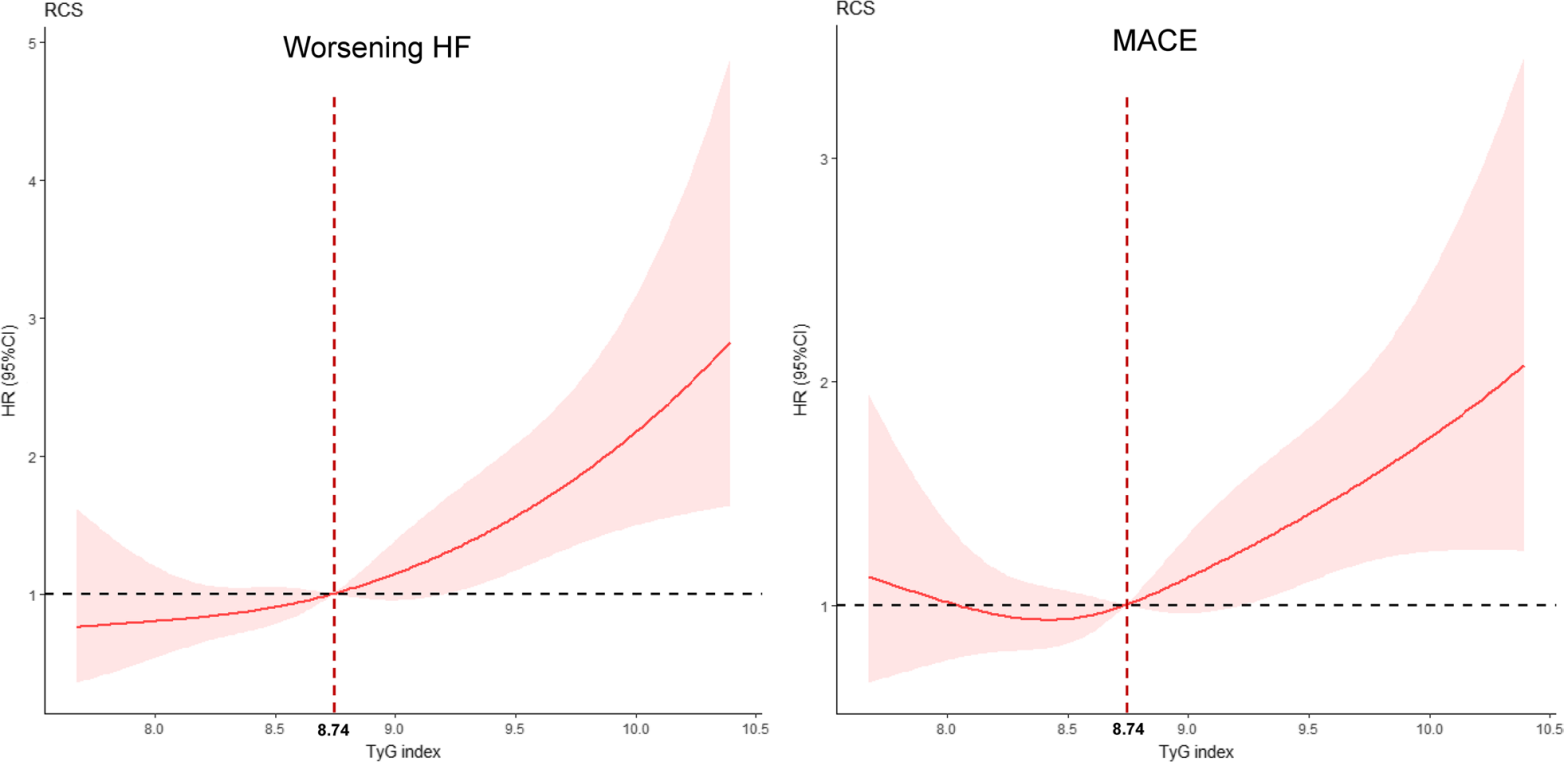
Restricted cubic spline regression analysis of TyG index with worsening HF and MACE. Heavy central lines represent the estimated; adjusted hazard ratios, with shaded ribbons denoting 95% confidence intervals. TyG index 8.74 was selected as the reference level represented by the vertical dotted lines

The restricted cubic spline models showed that the risk of worsening HF and MACE increased with TyG index: a 0.1-unit increase in the index was associated with a 1.07-fold increase in the risk of worsening HF and a 1.05-fold increase in the risk of MACE (Fig. 2 and Table 2).

**Table 2:** Risk of incident outcomes for the TyG index

Events/no. at risk		Model 1^a^		Model 2^b^		Model 3^c^	
		HR (95% CI)	P value	HR (95% CI)	P value	HR (95% CI)	P value
Worsening HF							
Per 0.1 unit increase		1.03 (1.02–1.08)	< 0.001	1.06 (1.04–1.09)	< 0.001	1.07 (1.05–1.10)	< 0.001
Tertile 1	48/309	Ref.		Ref.		Ref.	
Tertile 2	48/306	1.04 (0.70–1.56)	0.838	1.09 (0.73–1.63)	0.684	1.31 (0.82–2.07)	0.249
Tertile 3	81/307	1.88 (1.32–2.69)	0.001	2.02 (1.41–2.90)	< 0.001	2.44 (1.59–3.72)	< 0.001
MACE							
Per 0.1 unit increase		1.03 (1.01–1.05)	0.005	1.04 (1.02–1.06)	< 0.001	1.05 (1.02–1.07)	< 0.001
Tertile 1	75/309	Ref.		Ref.		Ref.	
Tertile 2	70/306	1.03 (0.74–1.43)	0.856	1.03 (0.70–1.53)	0.871	1.06 (0.71–1.60)	0.773
Tertile 3	96/307	1.48 (1.10–2.01)	0.011	1.66 (1.22–2.26)	0.001	1.90 (1.31–2.76)	0.001

^a^Unadjusted

^b^Adjusted for age, gender, left ventricular ejection fraction

^c^Adjusted for age, gender, smoking history, body mass index, left ventricular ejection fraction, hyperlipidemia, hypertension, diabetes mellitus, anemia, chronic kidney disease, acute myocardial infarction and atrial fibrillation, and renin–angiotensin–aldosterone system inhibitor, beta-blockers, loop diuretics, and mineralocorticoid receptor antagonist

*CI* confidence interval, *HR* hazard ratio, *MACE* major adverse cardiovascular events, *Worsening HF* worsening heart failure

**Fig. 3:**
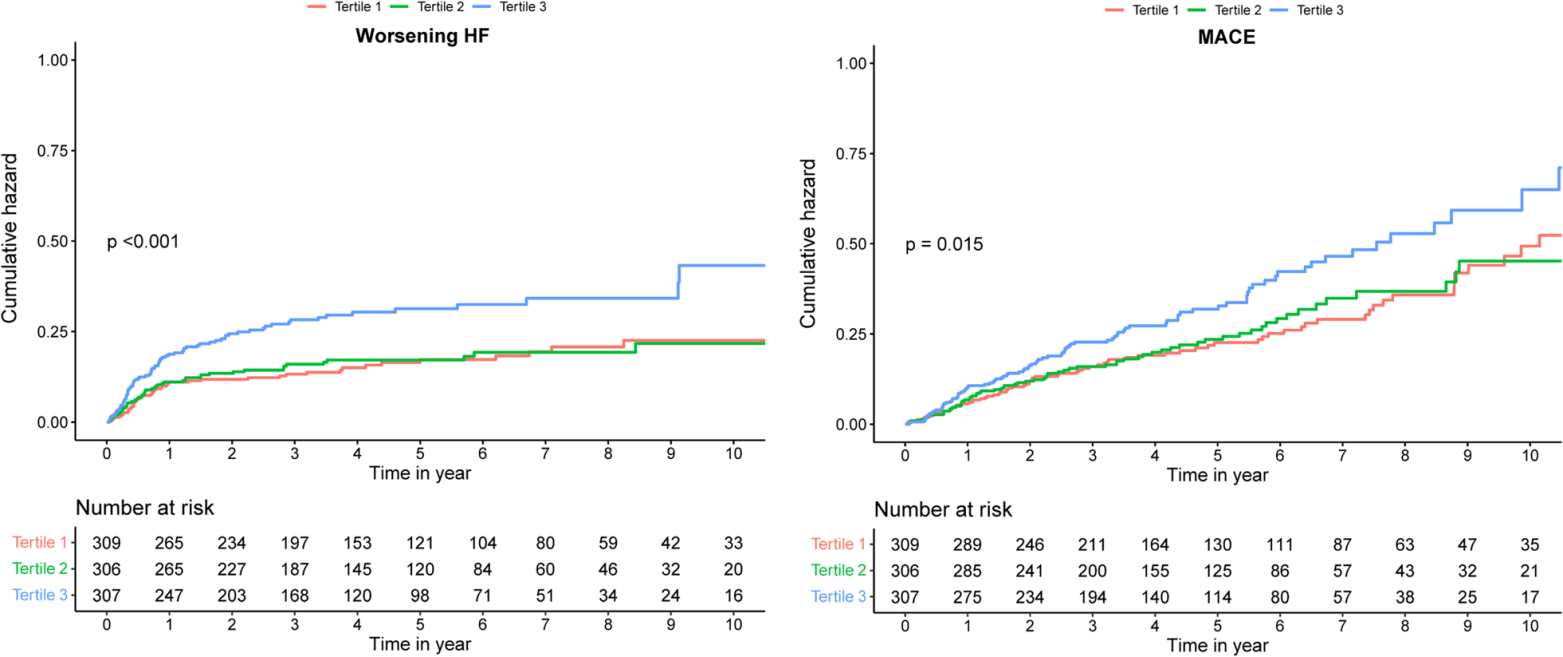
Kaplan–Meier survival curves based on TyG index tertile on significant sMR. **a** worsening HF; **b** major adverse cardiovascular events (MACE)

The median follow-up duration was 3.7 years (IQR 1.9–6.4 years). The multivariable analysis showed that the T3 group had a higher risk of worsening HF than the T1 group (26.4 vs 15.5%; adjusted HR = 2.44, 95% CI 1.59–3.72; P < 0.001). The Kaplan–Meier survival curve showed the same survival outcome (Fig. 3). The relationship between TyG index and MACE showed the same trends as the primary endpoint. Long-term MACE occurred in 31.3% of patients in the T3 group and 24.3% of those in the T1 group (adjusted HR = 1.90, 95% CI: 1.31–2.76; P < 0.001). The results of TG, FPG, and TG/FPG are shown in Additional file 1: Table S2.

### Subgroup analysis

**Fig. 4:**
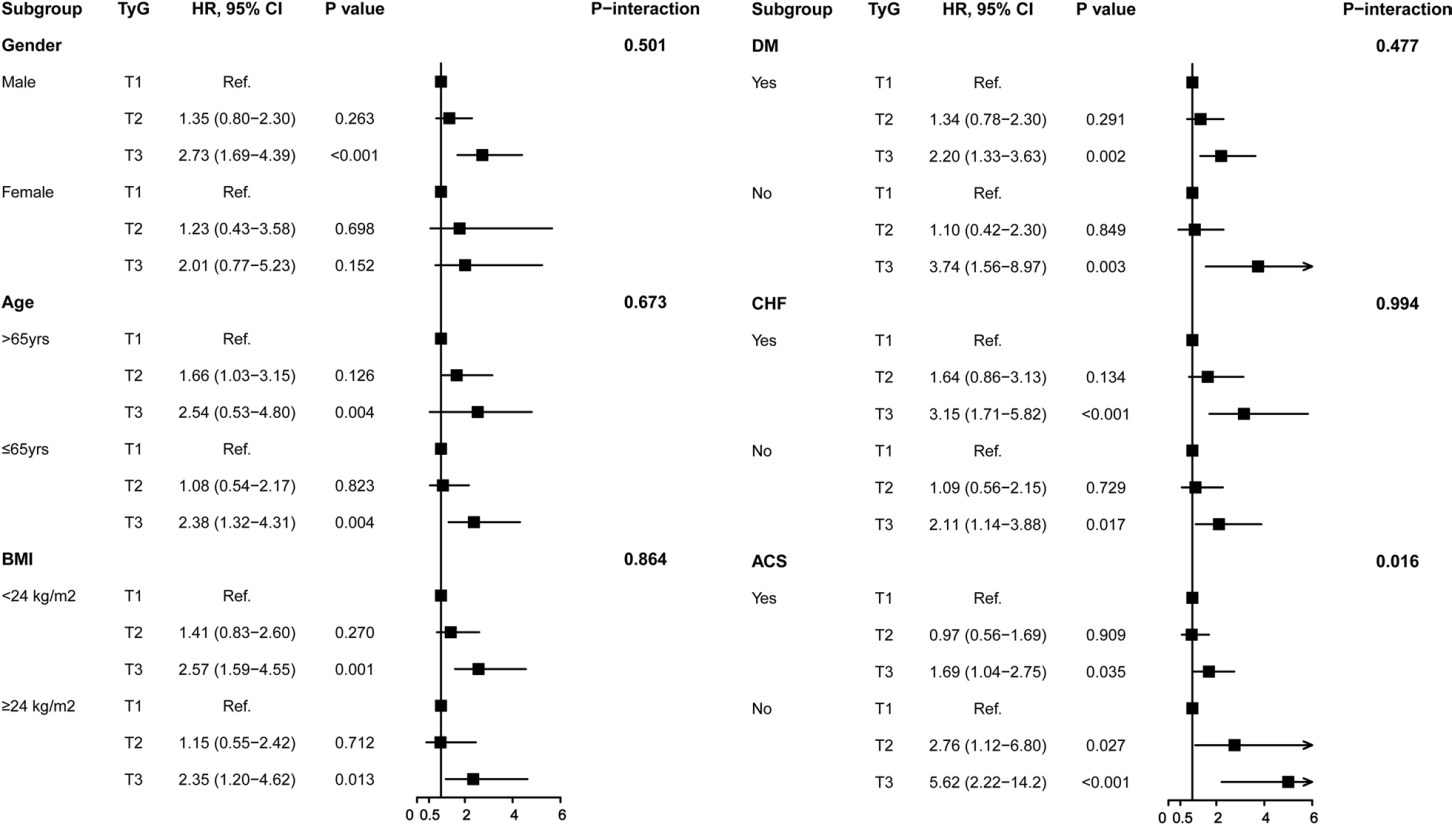
Subgroup and interaction analysis between the TyG index and worsening HF across various subgroups

The subgroup analysis showed that the association between the TyG index and risk of worsening HF was similar across patient subgroups stratified by age, gender, BMI, CHF, and DM (P values for interaction  > 0.05). A more significant trend of TyG index in predicting worsening HF was observed for stable CAD than for ACS (P value for interaction = 0.16; Fig. 4).

### Incremental effect of the TyG index on prediction of the primary endpoint

NRI, IDI, and the difference in C statistic were calculated for both primary and secondary endpoints by adding TyG index to clinical indices and models (Table 3). Including TyG index significantly improved risk prediction for the primary endpoint of worsening HF across all clinical indices and models. However, the predictive value of these models for MACE was not improved after adding TyG index, and adding TG, FPG, and TG/FPG did not significantly improve the predictive value of the clinical indices and models (Additional file 1: Table S3).

**Table 3:** Improvement in risk prediction by adding TYG index to risk factors and clinical models

	NRI [95% CI]	P value	IDI [95% CI]	P value	Δ in C-statistics	P value
5-year worsening HF						
ProBNP	0.19 (0.03–0.29)	< 0.001	0.02 (0.01–0.06)	< 0.001	0.01	< 0.001
LVEF	0.21 (0.06–0.31)	0.01	0.02 (0.00–0.05)	0.01	0.05	< 0.001
hs-cTnT	0.19 (0.01–0.28)	0.01	0.02 (0.01–0.05)	< 0.001	0.03	< 0.001
MUSIC Risk score	0.24 (0.06–0.33)	< 0.001	0.03 (0.00–0.06)	< 0.001	0.03	< 0.001
5-year MACE						
ProBNP	0.09 (− 0.03–0.18)	0.09	0.01 (0.00–0.03)	0.03	0.01	0.03
LVEF	0.07 (− 0.03–0.16)	0.189	0.01 (0.00–0.02)	0.06	0.02	0.03
hs-cTnT	0.05 (− 0.05–0.16)	0.289	0.01 (0.00–0.03)	0.109	0.04	0.04
GRACE score in ACS	0.06 (− 0.06–0.17)	0.299	0.01 (− 0.00–0.03)	0.100	0.00	0.105

*Δ* difference, *IDI* integrated discrimination improvement, *NRI* net reclassification index

## Discussion

To our knowledge, this is the first study to systematically assess the association between TyG index and the risk of worsening HF among patients with significant sMR after PCI. The main findings of the study were as follows: (i) TyG index was an independent predictor of adverse events in patients with significant sMR after PCI; (ii) TyG index is a simple and effective alternative measure to IR for stratifying patients with significant sMR after PCI according to risk of worsening HF; and (iii) adding TyG to risk factors and clinical models could improve the prediction of worsening HF.

Epidemiologic data show that moderate or severe sMR is a common reason for hospital admission and contributes to significant disease burden [22–24]. The prevalence of functional ischemic MR among CAD patients has been reported to be as high as 50%, and moderate or severe sMR accounts for nearly one-fifth of ischemic MR cases. The causes of sMR in patients with CAD are heterogeneous, which complicates prognostic assessment [5, 22, 25–27]. Many studies have shown that PCI, which is one of the most common methods of reperfusion and improves patient outcomes, can reduce the area of myocardial ischemia and reflux in sMR during follow-up [28, 29]. sMR is dynamic and patients may experience progressive HF despite PCI, which can be easily missed by clinicians. Therefore, prognostic assessment is important in the follow-up of CAD patients and for planning interventions [3, 30], and there is an urgent need for novel biomarkers that can be used predict outcomes of patients with significant sMR.

IR, which is caused by impaired glucose uptake and utilization, is a major feature of diabetes and risk factor for CVD [31, 32]. IR contributes to the development of CVD in both the general population and in patients with diabetes, and predicts cardiovascular events in patients with CVD [33]; it is also an indicator of HF and heart function deterioration [34, 35]. LV dysfunction is a major driver of sMR: one community cohort study found that sMR was associated with frequent post-diagnosis HF and increased mortality [36]. As such, identification of IR may have great clinical significance for improving cardiovascular risk stratification in significant sMR patients. The TyG index, as the product of FPG and triglycerides, is a novel index that has been suggested as a simple and reliable surrogate of IR and has been shown to be superior to homeostasis model assessment in predicting. [37] Numerous clinical studies have shown that the TyG index is correlated with the risk of incident CVD. Healthy participants with an elevated TyG index were found to be at higher risk of cardiovascular events [38]; and a cross-sectional observational study showed that an elevated TyG index was correlated with the incidence and severity of coronary stenosis in patients with DM [39]. The TyG index was also an independent predictor of progressive coronary artery calcification [40]. A high TyG index was associated with an increased risk of subclinical myocardial injury [41] and predicted the risk of MACE in patients with CAD [10, 11], ACS and DM [13, 42], ACS undergoing PCI [43], and HF [14, 15]. However, the predictive value of the TyG index for significant sMR has not been previously reported.

In this study, we investigated the relationship between the TyG index and worsening HF in patients with significant sMR. The selection of worsening HF as the primary endpoint was an important consideration and was based on the following findings from previous studies. TyG index was shown to be positively associated with HF risk in a J-shaped dose–response relationship in a Chinese community cohort [44]. Another study reported an association between the TyG index and a composite endpoint of cardiovascular death or rehospitalization for HF in patients with CHF and type 2 diabetes [15]. Meanwhile, the severity of sMR was significantly associated with HF events [36]. We defined worsening HF as unplanned rehospitalization or unscheduled physician office/emergency department visits due to HF or unplanned mitral valve surgery according to the characteristics of sMR patients. A higher TyG index was significantly associated with a higher risk of worsening HF, which persisted after adjusting for traditional cardiovascular risk factors, comorbidity burden, disease severity, and medications. Additionally, after adjustment, the TyG index was still an independent predictor of MACE although it did not improve the predictive ability of clinical models for MACE. On the other hand, by adding the TyG index to established risk factors for worsening HF, there was a significant improvement in risk prediction in terms of the NRI, IDI, and C statistic, and the incremental value of the TyG index was superior to that of FPG or fasting TG alone. Although a higher TyG index is known to be associated with worse cardiovascular outcomes, its incremental prognostic value has not been previously investigated. Our results suggest that the TyG index can refine cardiovascular risk stratification; routinely including the index in clinical diagnostic models can more accurately identify patients with high cardiovascular risk, thereby allowing more targeted treatment or implementation of preventative measures. Thus, our findings are in line with those of earlier studies demonstrating the clinical value of the TyG index in CVD patients, including those with significant sMR.

There are a few possible mechanisms linking the TyG index with worsening HF and MACE in patients with significant sMR. First, IR worsens LV remodeling and function and may promote myocardial hypertrophy and fibrosis via multiple signaling pathways including protein kinase B (Akt), transforming growth factor (TGF)-β, and peroxisome proliferator-activated receptor (PPAR)-γ [45]. IR can also induce changes in substrate metabolism and reduce the efficiency of energy metabolism, thereby impeding the normal myocardial response to injury [46]. Additionally, the metabolic efficiency of the myocardium is further reduced in patients through the downregulation of factors that regulate the β-oxidation of fatty acids [47]. Second, IR can cause abnormalities in subcellular components by inducing lipotoxicity, oxidative stress, mitochondrial dysfunction, endoplasmic reticulum stress, and impaired calcium signaling [6, 47]. Third, IR can induce immune cell infiltration into adipose tissue and macrophage activation; the secretion of proinflammatory mediators by immune cells and activated macrophages leads to low-grade local and systemic inflammation [6]. The vicious cycle of these conditions contributes to cardiomyocyte injury and death and cardiac hypertrophy and fibrosis [6]. Finally, cardiovascular calcification is a tightly regulated process similar to bone formation, and histopathologic and molecular similarities have been demonstrated between vascular atherosclerotic and valvular calcification [48]. The systemic inflammatory milieu of IR promotes procalcific mechanisms. Therefore, examining the link between significant sMR and IR can provide insights that aid risk stratification in patients with significant sMR and help to determine the appropriate time for intervention. Targeted analyses of risk factors and novel prediction tools for early identification and intervention before worsening HF are needed for PCI patients with significant sMR; the TyG index has clinical applicability in these regards.

For PCI populations with significant sMR, targeted analyses for related risk factors and further prediction tools to enable early identification and positive intervention before worsening HF are warranted. Evaluating the TyG index may provide early intervention in these patients and improve prognosis.

### Study limitations

There were several limitations to this study. First, sMR has many etiologies. The main goal of our study was to investigate the prognostic value of the TyG index in patients with significant sMR following PCI, but it is important to examine other sMR populations. Second, because of the observational nature of this study, there were likely unmeasured confounding factors related to prognosis; therefore, our results should be considered as hypothesis-generating, which limits their generalizability.

## Conclusion

The results of this study demonstrate that the TyG index is directly correlated with poor prognosis in patients with significant sMR following PCI. Thus, an elevated TyG index can serve as a predictor and risk stratification tool for worsening HF, although further prospective, large-scale studies are needed to confirm our findings.

## Supplementary Information

**Additional file 1:** **Table S1. **Baseline Characteristics According to TyG index level using Corrected p-value. **Table S2. **Risk of incident outcomes for the TG, FPG and TG/FPG. **Table S3. **Improvement in Risk Prediction by Adding TG, FPG and TG/FPG to risk factors and clinical models.

## Data Availability

Data are available from the corresponding author on reasonable request.
