# Supplementary material for: Association between triglyceride glucose index and worsening heart failure in significant secondary mitral regurgitation following percutaneous coronary intervention
Source: Cardiovasc Diabetol. 2022 Nov 28;21:260. doi: 10.1186/s12933-022-01680-9 (PMC9706938; doi:10.1186/s12933-022-01680-9)
Supplement: Supplementary file 1 — Additional file 1: Table S1. Baseline Characteristics According to TyG index level using Corrected p-value. Table S2. Risk of incident outcomes for the TG, FPG and TG/FPG. Table S3. Improvement in Risk Prediction by Adding TG, FPG and TG/FPG to risk factors and clinical models. [file 12933_2022_1680_MOESM1_ESM.docx]

**Additional file 1: Table 1 Baseline Characteristics According to TyG index level using Corrected p-value**

| **Characteristic** | **TyG index level** | | | | **Corrected p-value*** |
| --- | --- | --- | --- | --- | --- |
|  | **Overall** | **T1 (<8.51)** | **T2 (≥8.51, <8.98** | **T3 (≥8.98)** |  |
|  | **N= 922** | **N = 309** | **N = 306** | **N = 307** |  |
| **Demographic characteristics** | | |  |  |  |
| Male | 734 (79.6) | 261 (84.5) | 240 (78.4) | 233 (75.9) | 0.125 |
| Age, yrs | 64.1 (11.0) | 64.3 (11.4) | 65.4 (11.0) | 62.6 (10.5) | 0.018 |
| BMI, Kg/m^2^ | 23.8 (3.4) | 23.5 (3.4) | 23.5 (3.3) | 24.2 (3.6) | 0.063 |
| Heart rate, bmp | 82.1 (17.4) | 81.0 (16.6) | 81.3 (18.6) | 84.0 (16.7) | 0.177 |
| SBP, mmHg | 128.1 (22.8) | 126.9 (22.6) | 128.7 (22.1) | 128.6 (23.7) | NA |
| DBP, mmHg | 75.6 (12.9) | 75.5 (13.4) | 75.5 (12.2) | 75.7 (13.2) | NA |
| History of smoke | |  |  |  | 0.125 |
| Never | 567 (61.5) | 185 (59.9) | 180 (58.8) | 202 (65.8) |  |
| Cessation | 139 (15.1) | 59 (19.1) | 49 (16.0) | 31 (10.1) |  |
| Current | 216 (23.4) | 65 (21.0) | 77 (25.2) | 74 (24.1) |  |
| **Medical history** | |  |  |  |  |
| Anemia | 404 (43.8) | 145 (46.9) | 134 (43.8) | 125 (40.7) | NA |
| CHF | 416 (45.1) | 131 (42.4) | 139 (45.4) | 146 (47.6) | NA |
| DM | 404 (43.8) | 145 (46.9) | 134 (43.8) | 125 (40.7) | <0.001 |
| CKD | 341 (37.0) | 100 (32.4) | 112 (36.6) | 129 (42.0) | NA |
| Hypertension | 552 (59.9) | 183 (59.2) | 183 (59.8) | 186 (60.6) | NA |
| Hyperlipidemia | 690 (74.8) | 226 (73.1) | 233 (76.1) | 231 (75.2) | NA |
| AF | 105 (11.4) | 52 (16.8) | 26 (8.5) | 27 (8.8) | 0.003 |
| COPD | 29 (3.1) | 10 (3.2) | 11 (3.6) | 8 (2.6) | NA |
| Stroke | 39 (4.2) | 15 (4.9) | 12 (3.9) | 12 (3.9) | NA |
| History of PCI | 119 (12.9) | 40 (12.9) | 38 (12.4) | 41 (13.4) | NA |
| History of AMI | 99 (10.7) | 29 (9.4) | 31 (10.1) | 39 (12.7) | NA |
| Clinical presentation | |  |  |  |  |
| AMI | 343 (37.2) | 101 (32.7) | 116 (37.9) | 126 (41.0) | 0.285 |
| STEMI | 227 (24.6) | 72 (23.3) | 75 (24.5) | 80 (26.1) | NA |
| NSTEMI | 116 (12.6) | 29 (9.4) | 41 (13.4) | 46 (15.0) | 0.291 |
| **Laboratory test** | |  |  |  |  |
| LDL-C, mmol/L | 2.88 (1.10) | 2.77 (1.05) | 2.95 (1.19) | 2.91 (1.06) | 0.351 |
| HDL-C, mmol/L | 0.97 (0.27) | 0.98 (0.28) | 0.97 (0.27) | 0.96 (0.28) | NA |
| eGFR, mL/min/1.73m2 | 69.6 (26.0) | 73.5 (27.4) | 68.6 (23.9) | 66.8 (26.1) | 0.012 |
| ALB, g/L | 35.0 (4.6) | 34.5 (4.1) | 35.3 (5.1) | 35.1 (4.5) | 0.264 |
| NT-proBNP, ng/L | 2092 [903, 4826] | 2414 [1169, 5311] | 1864 [860, 4606] | 1739 [749, 4816] | 0.12 |
| hs-cTnT, ng/L | 0.71 [0.22, 7.33] | 0.64 [0.22, 4.20] | 0.62 [0.22, 6.33] | 1.13 [0.23, 9.89] | 0.507 |
| LVEF, % | 43.8 (13.7) | 42.8 (13.6) | 45.5 (13.9) | 43.2 (13.4) | 0.087 |
| LVEDD, mm | 43.3 (10.7) | 44.1 (10.8) | 42.5 (10.6) | 43.3 (10.5) | 0.495 |
| LVESD, mm | 56.6 (8.5) | 57.3 (8.7) | 56.1 (8.5) | 56.5 (8.4) | 0.66 |
| Left atrial, mm | 41.2 (6.3) | 41.7 (6.3) | 41.0 (6.7) | 41.1 (6.0) | 0.927 |
| **Procedural characteristics** | |  |  |  |  |
| Radial artery access | 754 (81.8) | 256 (82.8) | 247 (80.7) | 251 (81.8) | NA |
| Multivessel disease | 789 (85.6) | 263 (85.1) | 261 (85.3) | 265 (86.3) | NA |
| Culprit vessel in STEMIs | |  |  |  | NA |
| Left main coronary artery | 7 (2.8) | 1 (1.4) | 3 (3.6) | 3 (3.4) |  |
| LAD | 115 (46.6) | 37 (50.0) | 39 (46.4) | 39 (43.8) |  |
| LCX | 41 (16.6) | 11 (14.9) | 13 (15.5) | 17 (19.1) |  |
| RCA | 84 (34.0) | 25 (33.8) | 29 (34.5) | 30 (33.7) |  |
| Number of vessels treated | 1.45 (0.68) | 1.41 (0.67) | 1.49 (0.68) | 1.44 (0.69) | NA |
| Number of lesions treated | 1.67 (0.89) | 1.63 (0.89) | 1.70 (0.86) | 1.68 (0.94) | NA |
| Number of stents | 1.91 (1.18) | 1.92 (1.16) | 1.92 (1.24) | 1.90 (1.15) | NA |
| Minimum stent diameter, mm | 2.69 (0.75) | 2.70 (0.77) | 2.64 (0.80) | 2.72 (0.69) | NA |
| Lesion length, mm | 52.3 (36.3) | 52.5 (36.0) | 52.5 (38.4) | 52.0 (34.7) | NA |
| **Discharge prescription** | |  |  |  |  |
| RAAS inhibitor | 618 (67.0) | 220 (71.2) | 191 (62.4) | 207 (67.4) | 0.201 |
| Beta-blockers | 781 (84.7) | 265 (85.8) | 254 (83.0) | 262 (85.3) | NA |
| CCB | 144 (15.6) | 40 (12.9) | 47 (15.4) | 57 (18.6) | 0.468 |
| Statin | 856 (92.8) | 287 (92.9) | 286 (93.5) | 283 (92.2) | NA |
| Aspirin | 870 (94.4) | 293 (94.8) | 285 (93.1) | 292 (95.1) | NA |
| Clopidogrel | 807 (87.5) | 276 (89.3) | 267 (87.3) | 264 (86.0) | NA |
| Loop diuretic | 461 (50.0) | 154 (49.8) | 149 (48.7) | 158 (51.5) | NA |
| MRA | 467 (50.7) | 160 (51.8) | 149 (48.7) | 158 (51.5) | NA |
| Hypoglycemic drugs | 199 (21.9) | 40 (13.0) | 67 (22.3) | 92 (30.7) | <0.001 |
| Insulin | 40 (4.3) | 10 (3.2) | 10 (3.3) | 20 (6.5) | 0.216 |

*Bonferroni-corrected p value was calculated as the each p-value multiplied by the number of tests (n = 3).

**Abbreviation:** AF = atrial fibrillation; AMI = acute myocardial infarction; ALB = albumin; CHF = congestive heart failure; CKD = chronic kidney disease; COPD = chronic obstructive pulmonary disease; CCB = calcium channel blockers; DBP = diastolic blood pressure; DM = diabetes; eGFR = estimated glomerular filtration rate; HDL-C = high-density lipoprotein cholesterol; hs-cTnT = Hypersensitive troponin T; LDL-C = low-density lipoprotein cholesterol; LAD = left anterior descending coronary artery; LCX = left circumflex coronary artery; LVEF, left ventricular ejection fraction; LVEDD, left ventricular end-diastolic dimension; LVESD, left ventricular end-systolic dimension; MRA = mineralcorticoid recept antagonist; NSTEMI = non-ST-segment elevation myocardial infarction; NT-proBNP = N-terminal pro brain natriuretic peptide; Triglyceride–glucose index = TyG index; PCI = percutaneous coronary intervention; RAAS inhibitor = renin-angiotensin-aldosterone system inhibitor; RCA = right coronary artery; SBP = systolic blood pressure; STEMI = ST-segment elevation myocardial infarction.

**Additional file 1:** Table 2 Risk of incident outcomes for the cumulative TG, FPG and TG/FPG.

| **Worsening HF** | Model 1 ^a^ | | Model 2 ^b^ | | | | Model 3 ^c^ | |  |
| --- | --- | --- | --- | --- | --- | --- | --- | --- | --- |
|  | HR (95% CI) | P Value | | HR (95% CI) | P Value | HR (95% CI) | | P Value | |
| **TG** |  |  | |  |  |  | |  | |
| Per 0.1 Unit increase | 1.01 (0.99-1.02) | 0.228 | | 1.01 (0.99-1.03) | 0.07 | 1.02 (1.00-1.03) | | 0.04 | |
| Tertile 1 | Ref. |  | | Ref. |  | Ref. | |  | |
| Tertile 2 | 0.90 (0.62-1.32) | 0.598 | | 0.99 (0.68-1.45) | 0.958 | 0.99 (0.59-1.38) | | 0.626 | |
| Tertile 3 | 1.25 (0.88-1.77) | 0.217 | | 1.44 (1.00-2.06) | 0.048 | 1.53 (1.02-2.29) | | 0.039 | |
| **FPG** |  |  | |  |  |  | |  | |
| Per 0.1 Unit increase | 1.01 (1.00-1.01) | <0.001 | | 1.01 (1.00-1.01) | 0.012 | 1.01 (1.00-1.01) | | 0.008 | |
| Tertile 1 | Ref. |  | | Ref. |  | Ref. | |  | |
| Tertile 2 | 0.91 (0.63-1.32) | 0.615 | | 0.91 (0.60-1.37) | 0.643 | 0.91 (0.59-1.38) | | 0.845 | |
| Tertile 3 | 1.54 (1.04-2.29) | 0.033 | | 1.54 (0.99-2.41) | 0.057 | 1.54 (0.97-2.45) | | 0.064 | |
| **TG/FPG** |  |  | |  |  |  | |  | |
| Per 0.1 Unit increase | 0.88 (0.79-0.99) | 0.036 | | 0.91 (0.82-1.03) | 0.123 | 0.88 (0.77-1.01) | | 0.075 | |
| Tertile 1 | Ref. |  | | Ref. |  | Ref. | |  | |
| Tertile 2 | 0.95 (0.68-1.34) | 0.784 | | 1.02 (0.72-1.44) | 0.909 | 0.95 (0.65-1.38) | | 0.768 | |
| Tertile 3 | 0.63 (0.44-0.92) | 0.018 | | 0.71 (0.49-1.05) | 0.085 | 0.65 (0.42-1.01) | | 0.06 | |

^a^ unadjusted

^b^ adjusted for age, gender, left ventricular ejection fraction;

^c^ adjusted for age, gender, smoking history, body mass index, left ventricular ejection fraction, hyperlipidemia, hypertension, diabetes mellitus, anemia, chronic kidney disease, acute myocardial infarction and atrial fibrillation, and renin-angiotensin-aldosterone system inhibitor, beta-blockers, loop diuretics, and mineralocorticoid receptor antagonist

CI = confidence interval; HR = Hazard Ratio; MACE = major adverse cardiovascular events; Worsening HF = worsening heart failure;

**Additional file 1: Table 3 Improvement in Risk Prediction by Adding TG, FPG and TG/FPG to risk factors and clinical models**

| **Worsening HF** | NRI [95% CI] | P Value | IDI [95% CI] | P Value | Δ in C-Statistics | P Value |
| --- | --- | --- | --- | --- | --- | --- |
| TG |  |  |  |  |  |  |
| ProBNP | 0.11 (-0.08-0.19 | 0.139 | 0.00 (-0.01-0.02) | 0.08 | 0.01 | 0.152 |
| LVEF | 0.10 (-0.04-0.18) | 0.149 | 0.00 (0.00-0.02) | 0.149 | 0.01 | 0.158 |
| hs-cTnT | 0.08 (-0.04-0.17) | 0.189 | 0.00 (0.00-0.02) | 0.199 | 0.02 | 0.176 |
| MUSIC Risk score | 0.10 (-0.10-0.20) | 0.259 | 0.00 (-0.01-0.02) | 0.229 | 0.01 | 0.172 |
| FPG |  |  |  |  |  |  |
| ProBNP | 0.06 (-0.05-0.16) | 0.229 | 0.00 (0.00-0.01) | 0.259 | 0.02 | 0.08 |
| LVEF | 0.04 (-0.08-0.13) | 0.388 | 0.01 (0.00-0.02) | 0.149 | 0.02 | 0.03 |
| hs-cTnT | 0.10 (-0.01-0.20) | 0.08 | 0.01 (0.00-0.02) | 0.06 | 0.06 | 0.04 |
| MUSIC Risk score | 0.01 (-0.20-0.14) | 0.597 | 0.00 (0.00-0.01) | 0.299 | 0.01 | 0.08 |
| TG/FPG |  |  |  |  |  |  |
| ProBNP | 0.05 (-0.05-0.16) | 0.219 | 0.00 (0.00-0.01) | 0.358 | -0.02 | 0.03 |
| LVEF | 0.05 (-0.05-0.14) | 0.239 | 0.00 (0.00-0.02) | 0.139 | 0.01 | 0.07 |
| hs-cTnT | 0.09 (-0.02-0.19) | 0.1 | 0.00 (0.00-0.02) | 0.06 | 0.03 | 0.05 |
| MUSIC Risk score | 0.06 (-0.07-0.15) | 0.279 | 0.00 (0.00-0.02) | 0.169 | 0.01 | 0.03 |

Δ = difference; IDI = integrated discrimination improvement; NRI = net reclassification index; other abbreviations as in Table 1.
